# Efficient Hydrogen Generation From Hydrolysis of Sodium Borohydride in Seawater Catalyzed by Polyoxometalate Supported on Activated Carbon

**DOI:** 10.3389/fchem.2020.00676

**Published:** 2020-08-11

**Authors:** Senliang Xi, Xiaojun Wang, Dan Wu, Xingtao Hu, Shixue Zhou, Hao Yu

**Affiliations:** ^1^College of Chemical and Biological Engineering, Shandong University of Science and Technology, Qingdao, China; ^2^Research Center, Jiangsu Xinju Environmental Co., Ltd., Nantong, China; ^3^Department of Research, Taian Boao Safety Evaluation Co., Ltd., Tai'an, China

**Keywords:** hydrogen production, activated carbon, polyoxometalate, NaBH_4_, seawater

## Abstract

Phosphotungstic acid (HPW) as a polyoxometalate was selected as the active component of the catalyst. The activated carbon supported different percentage of HPW catalysts were prepared by impregnation and were characterized by X-ray diffraction (XRD), nitrogen adsorption, Fourier transform infrared (FTIR), and scanning electron microscope (SEM). The results showed that the HPW retained the original Keggin structure after being supported on activated carbon, the specific surface of the HPW/C was much bigger than that of pure HPW. The catalytic performance of HPW/C in the hydrogen generation reaction by hydrolysis of sodium borohydride in seawater and in deionized water were studied. 2.5 wt.% HPW/C showed the fastest hydrolysis reaction rate and the biggest volume of hydrogen generated. As for hydrolysis of sodium borohydride, catalytic effect of HPW/C was better in seawater than in distilled water. HPW dispersed on activated carbon is a real promising catalytic system for the development of hydrogen generation by hydrolysis of NaBH_4_ in seawater.

## Introduction

Hydrogen is considered as an important alternative energy source for fossil fuels due to its diverse sources, cleanliness, and renewable advantages (Akdim et al., 2009; Yu et al., 2014; Zhou et al., 2014; Sun et al., 2018). Metal borohydride can release hydrogen through a simple hydrolysis reaction. Sodium borohydride owing to its low cost and high hydrogen density (10.6 wt.%), has received extensive attention as a promising hydrogen storage medium (Demirci, 2018; Lale et al., 2018; Lee et al., 2019). NaBH_4_ hydrolysis produced hydrogen by the following chemical reaction:

$$\begin{array}{l} \text{NaB}{\text{H}}_{4}+2{\text{H}}_{2}\text{O}\to \text{NaB}{\text{O}}_{2}+4{\text{H}}_{2}\;\Delta \text{H}=-217\text{kJ}/\text{mol} \end{array}$$

However, this reaction rate is very slow at room temperature, an appropriate catalyst is usually used to accelerate the reaction rate. Noble metal catalysts have been the research focus due to their stable chemical properties and good catalytic activity, such as Pt (Genç et al., 2018), Pd (Lu et al., 2018), Ru (Semiz et al., 2018), and its alloys (Alasmar et al., 2018; Semiz et al., 2018). However, their expensiveness and finite reserves limit their wide application.

Therefore, many studies have focused on the preparation of low-cost, highly active non-noble metal catalysts (Liang et al., 2009; Bennici et al., 2011; Lee et al., 2019; Nuran et al., 2019), such as CoB (Wang et al., 2018; Nuran et al., 2019), NiB (Hua, 2003; Singh and Das, 2017), Co/Fe_3_O_4_@C (Chen et al., 2018) and so on. Compared with noble metal, these catalysts have the advantages of simpler preparation and lower catalyst cost. Nevertheless, there are also shortcomings such as long initial activation time and poor catalytic performance of the catalyst, which need to be further improved. The surface acid/base character of the catalyst is an important point to improve the catalytic performance (Akdim et al., 2009).

H_3_PW_12_O_40_ (HPW) is a kind of polyoxometalate with strong acid properties. However, due to the difficulty of catalytic recovery (Lana et al., 2006; Leão Lana et al., 2006),certain pollution and the corrosion of equipment, the specific surface area is small (<10 m^2^/g), which is not conducive to giving full play to its catalytic activity and limiting its application in catalysis. The effective loading of polyoxometalates on suitable support can greatly increase its surface area. Therefore, not only its catalytic activity and selectivity were improved, but also the product was easy to separate, and the catalyst was easily regenerated, the production process was simplified. There are many works studying polyoxometalate on a variety of supports (Hanif et al., 2017; Alcañiz-Monge et al., 2018; Yuan et al., 2020), the research on surface properties showed that supporting polyoxometalate firmly on the support was key (Hu et al., 1995). Activated carbon not only has high specific surface area and large pore size but also can be adjusted as needed; it was a good catalyst support.

This study aims to prepare activated carbon supported polyoxometalate catalysts and use them for the hydrolysis of sodium borohydride to produce hydrogen, due to the extremely rich seawater resources in coastal cities, seawater was used as a reaction liquid. Our work is to study the mode of action between polyoxometalate and activated carbon, and the effect of catalyst microstructure on hydrogen generated kinetics.

## Experimental Details

### Catalyst Preparation

Activated carbon particles (AR, ≥200 mesh) and phosphotungstic acid (HPW) (≥95%) were purchased from Aladdin. Activated carbon was activated by chemical activation using nitric acid as an activator (Salem and Ebrahim Yakoot, 2016; Yao et al., 2016; Jiang et al., 2017). HPW/C catalysts were prepared by impregnation-chemical reduction method: Added activated carbon pretreated to the HPW solution, stirred them in a water bath at 50°C for 6 h. After filtration and washed with distilled water, the catalysts were dried at 105°C for 10 h under vacuum oven. Adjust the mass ratio of activated carbon and HPW to prepare different catalysts: 2.5 wt.%HPW/C, 4 wt.%HPW/C, 6 wt.%HPW/C, 10 wt.%HPW/C, 12.5 wt.%HPW/C.

### Characterization of Catalysts

#### X-Ray Diffraction (XRD)

The catalysts were determined by XRD of Rigaku Utima IV (Rigaku Corporation, Japan) operating at a scanning speed of 5 deg/min from 10 to 80° and in steps of 0.02° with Cu Kα radiation (40 kV, 40 mA).

#### Specific Surface and Porosity

N_2_-physisorption measurements were performed at −196°C, using a Micromeritic ASAP2460 (Micromeritics Instrument Corporation, America). The specific surface, pore volume and pore size distribution of activated carbon and catalysts were obtained by using BET (Brunner–Emmet–Teller), BJH (Barrett-Joyner-Halenda), and t-plot modelizations, respectively.

#### Fourier Transform Infrared (FTIR)

The functional group vibration during the carbon raw material pretreatment were characterized by a 510P Fourier transform infrared spectrometer (Nicolet Corporation, America). The KBr tablet method was used, and the transmitted light wavelength range was 4000–400 cm^−1^.

### Scanning Electron Microscope (SEM) and Energy Dispersive X-Ray Spectroscopy (EDX)

Activated carbon and catalysts morphology were observed by using SEM performed on an APREO (FEI, America) operating at 2 kV. And the HPW loaded on activated carbon was detected by EDX of QUANTAX (BRUKER, Germany) operating at 10 kV. The samples were deposited onto conducting resin and treated by gold sputtering.

### Sodium Borohydride Hydrolysis

The reaction temperature was 40°C, and 50 ml seawater (taken from Jiaozhou Bay) was used as the reaction liquid, the amount of powder NaBH_4_ (granular, 99.99% trace metals basis, Sigma-Aldrich) was around 500 mg, and the m (catalyst):m (NaBH_4_) is about 1:10. The reaction was performed under strong stirring conditions. Immediately after that, the generated hydrogen was collected by the drainage method.

## Results and Discussion

### Materials Characterization

#### Morphology of Materials

**Figure 1:**
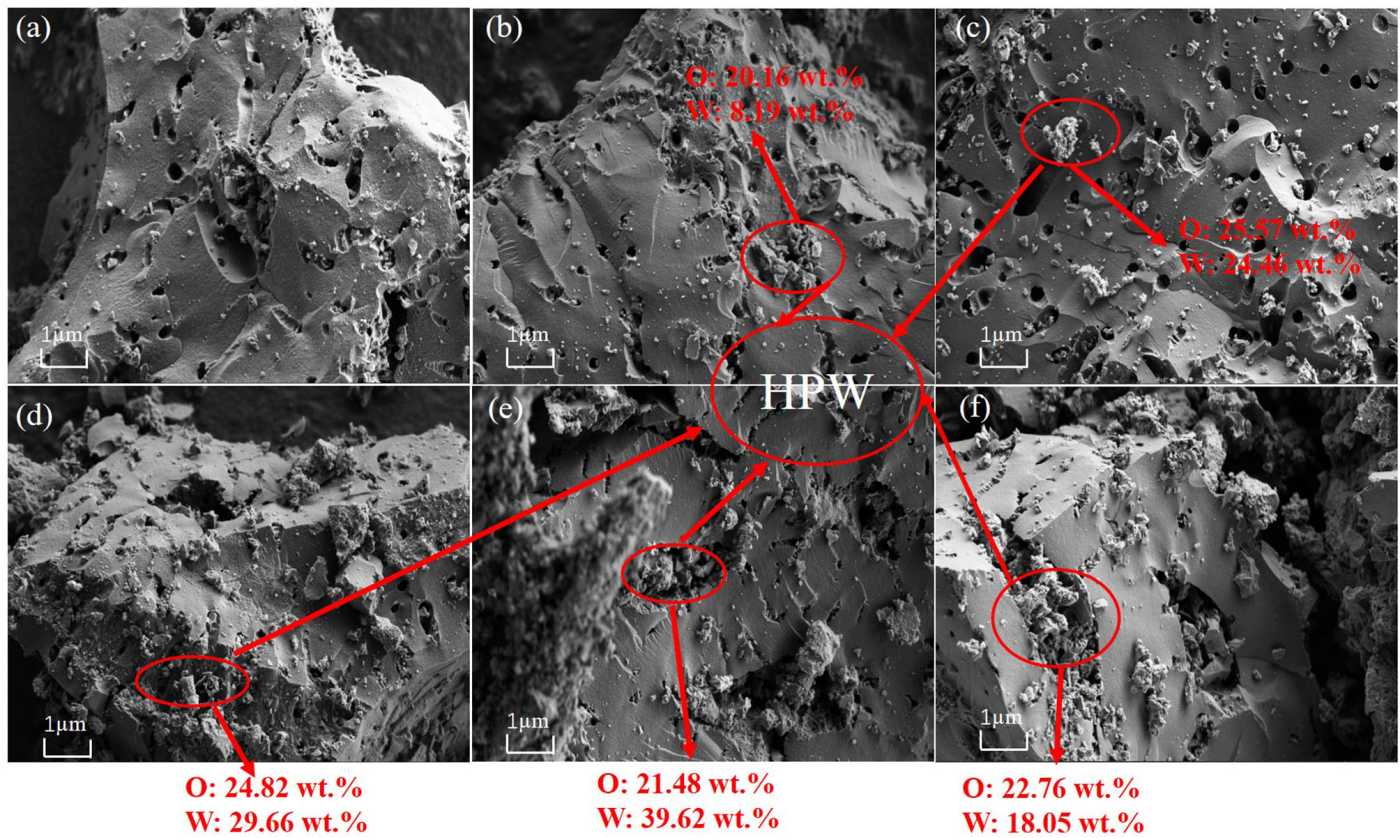
The SEM images and EDX detection of HPW with different loading percentages supported on the activated carbon: pure activated carbon **(a)**, 2.5 wt.%HPW/C **(b)**, 4 wt.%HPW/C **(c)**, 6 wt.%HPW/C **(d)**, 10 wt.%HPW/C **(e)**, 12.5 wt.%HPW/C **(f)**.

Figure 1 showed the SEM images of activated carbon and different catalysts samples with different HPW loadings. The HPW entered the pores of carbon increases the contact area of the catalyst with NaBH_4_ and reaction solution. Comparing Figures 1a–f, as the HPW loaded on the activated carbon, some HPW enters the pores of the activated carbon support, resulting in reducing the surface area and pore volume of activated carbon. Through EDX detection, the content of W and O confirmed the existence of HPW, and it can be seen that HPW entered the pores of activated carbon.

#### X-Ray Diffraction of Materials

**Figure 2:**
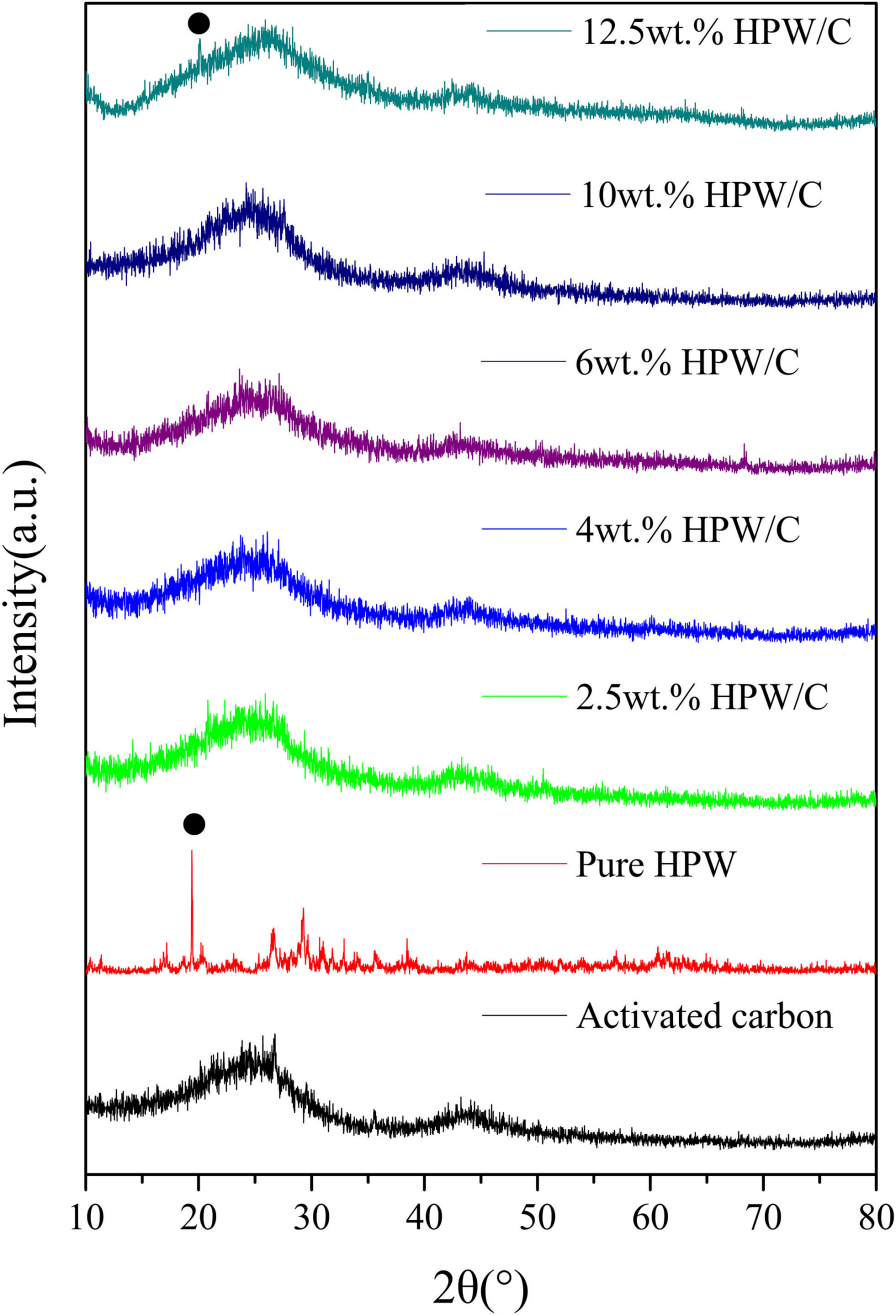
The XRD pattern of activated carbon, HPW and catalysts samples with different HPW loadings.

The X-ray diffraction pattern of each catalyst was shown in Figure 2. The XRD pattern can be used to study the crystal form of polyoxometalate on activated carbon (Song et al., 1996). The XRD pattern of the low-load catalyst was similar to the support, and no HPW crystal phase peak appeared. Only the high-load catalyst sample showed weak crystal phase diffraction peaks. This showed that the adsorption between activated carbon and HPW was not a simple physical action. The HPW adsorbed on the activated carbon had chemically bonded with the surface groups of the support. HPW was highly dispersed and contained oxygen on the support surface. The association was lost, and the original crystal form was lost, and the peak was not obvious. When the crystalline phase diffraction peaks appeared, HPW formed a bulk phase on the support surface, indicated that when the single molecule adsorption has reached saturation, the bulk phase polyoxometalate began to appear on the support surface.

#### Specific Surface Area and Pores

**Figure 3:**
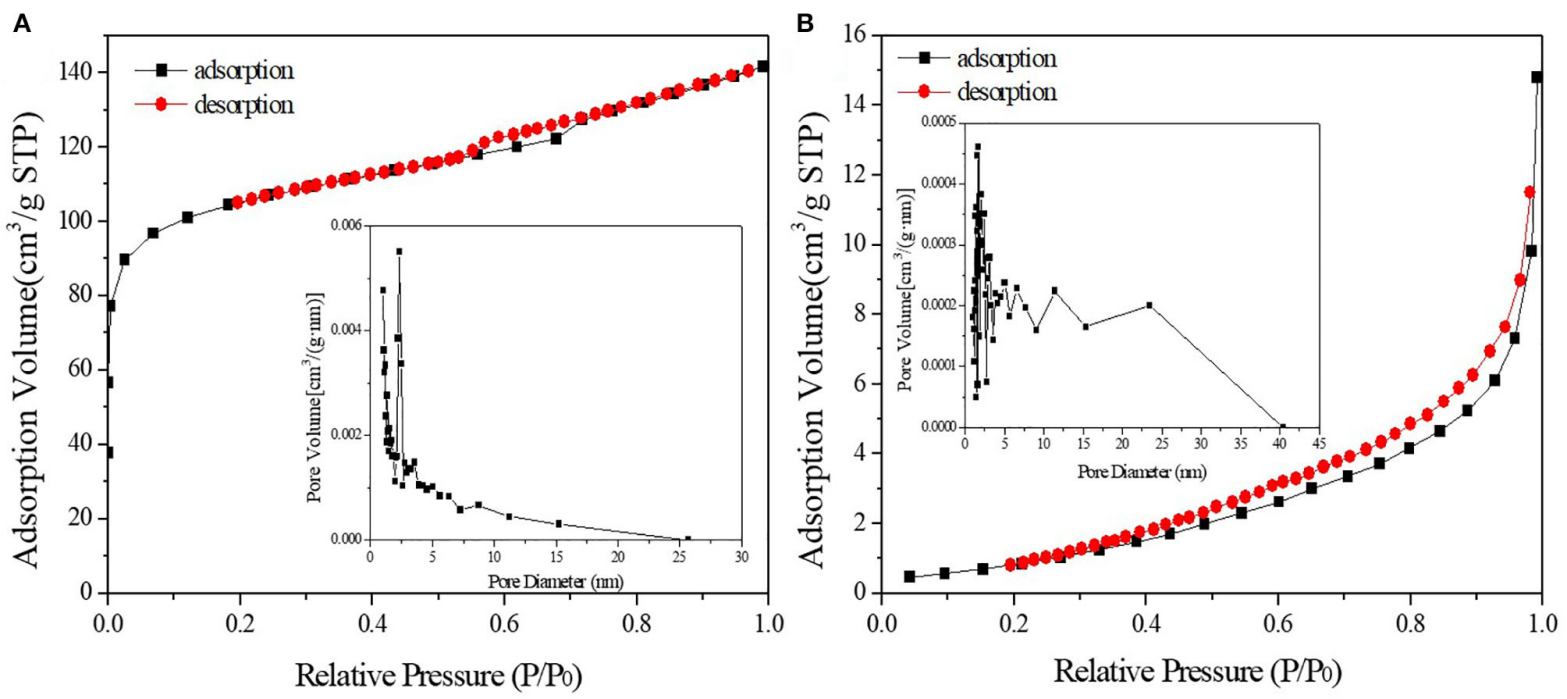
N_2_ adsorption/desorption isotherms and pore size distribution of activated carbon **(A)** and HPW **(B)**.

**Figure 4:**
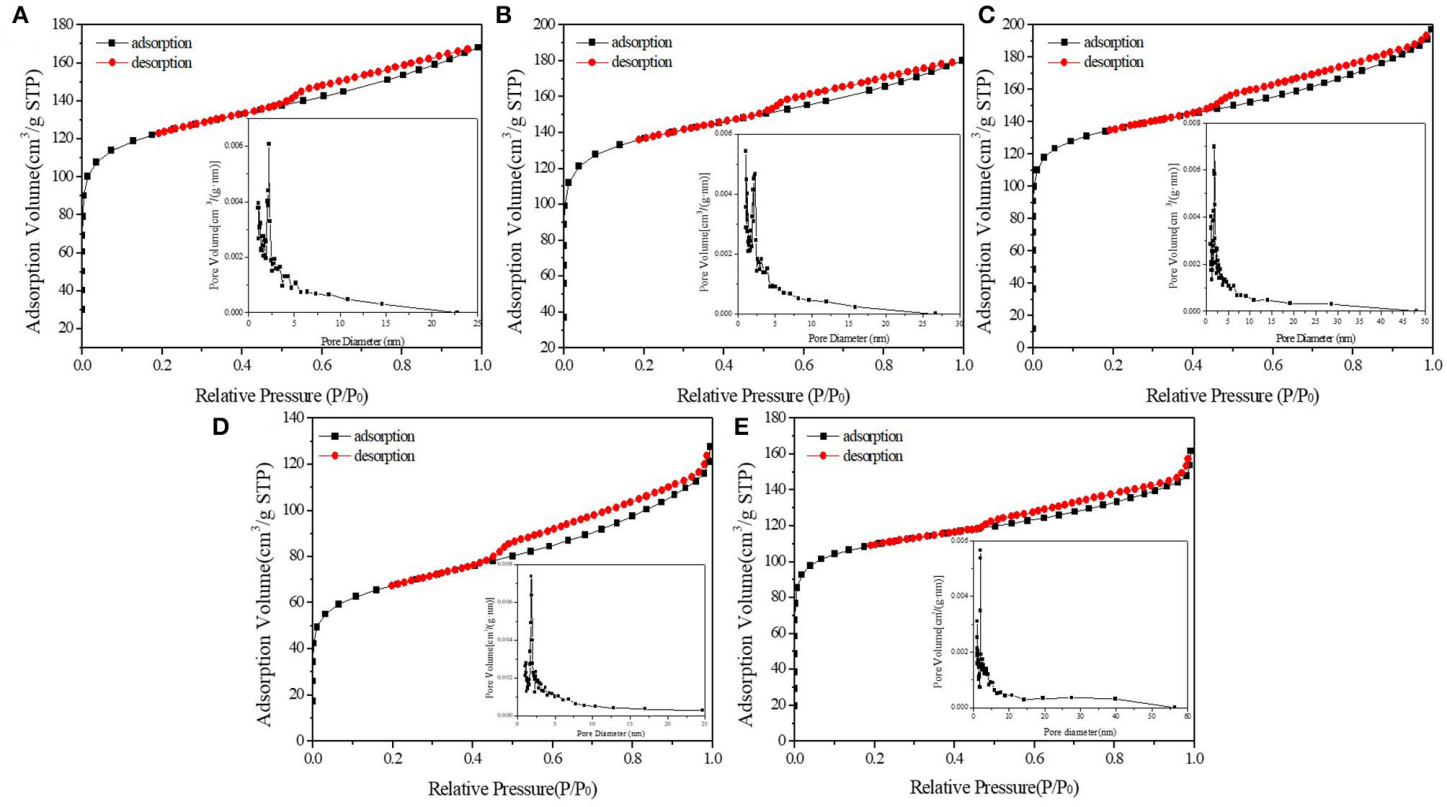
N_2_ adsorption/desorption isotherms and pore size distribution of 2.5 wt.%HPW/C **(A)**, 4 wt.%HPW/C **(B)**, 6 wt.%HPW/C **(C)**, 10 wt.%HPW/C **(D)**, 12.5 wt.%HPW/C **(E)**.

Figures 3, 4 demonstrated the N_2_ adsorption/desorption isotherms and pore size distribution of activated carbon, HPW, and different catalyst samples. The results in Table 1 showed that only the catalysts' specific surface area and pore volume value decreased rapidly with increasing HPW loading. Because the HPW filled the micropores and part of the mesopores of the activated carbon during the adsorption and diffusion process. Therefore, compared with pure HPW, the specific surface area of supported HPW was significantly increased, which was beneficial for catalytic reactions and effectively overcomes the lack of specific surface area of pure HPW when used as a catalyst. But 10 wt.%HPW/C showed a smaller surface area and pore volume, and a bigger average pore size than 12.5 wt.%HPW/C. It maybe because during the impregnation process, HPW caused partial micropore clogging. Therefore, in the nitrogen adsorption test, the blocked micropores cannot adsorb nitrogen, the specific surface area and pore volume were small, and the average pore diameter was large.

**Table 1:** Specific surface area, pore size and pore volume of activated carbon, HPW and catalysts samples with different HPW loadings.

**Samples**	**Surface area(m^**2**^/g)**	**Average pore diameter(nm)**	**Pore volume(cm^**3**^/g)**
Activated carbon	614.265	1.3	0.399
Pure HPW	4.051	11.37	0.023
2.5 wt.%HPW/C	449.351	1.43	0.321
4 wt.%HPW/C	444.087	1.42	0.313
6 wt.%HPW/C	444.603	1.38	0.307
10 wt.%HPW/C	234.582	1.69	0.199
12.5 wt.%HPW/C	360.768	1.39	0.251

#### FTIR Spectra Analysis

**Figure 5:**
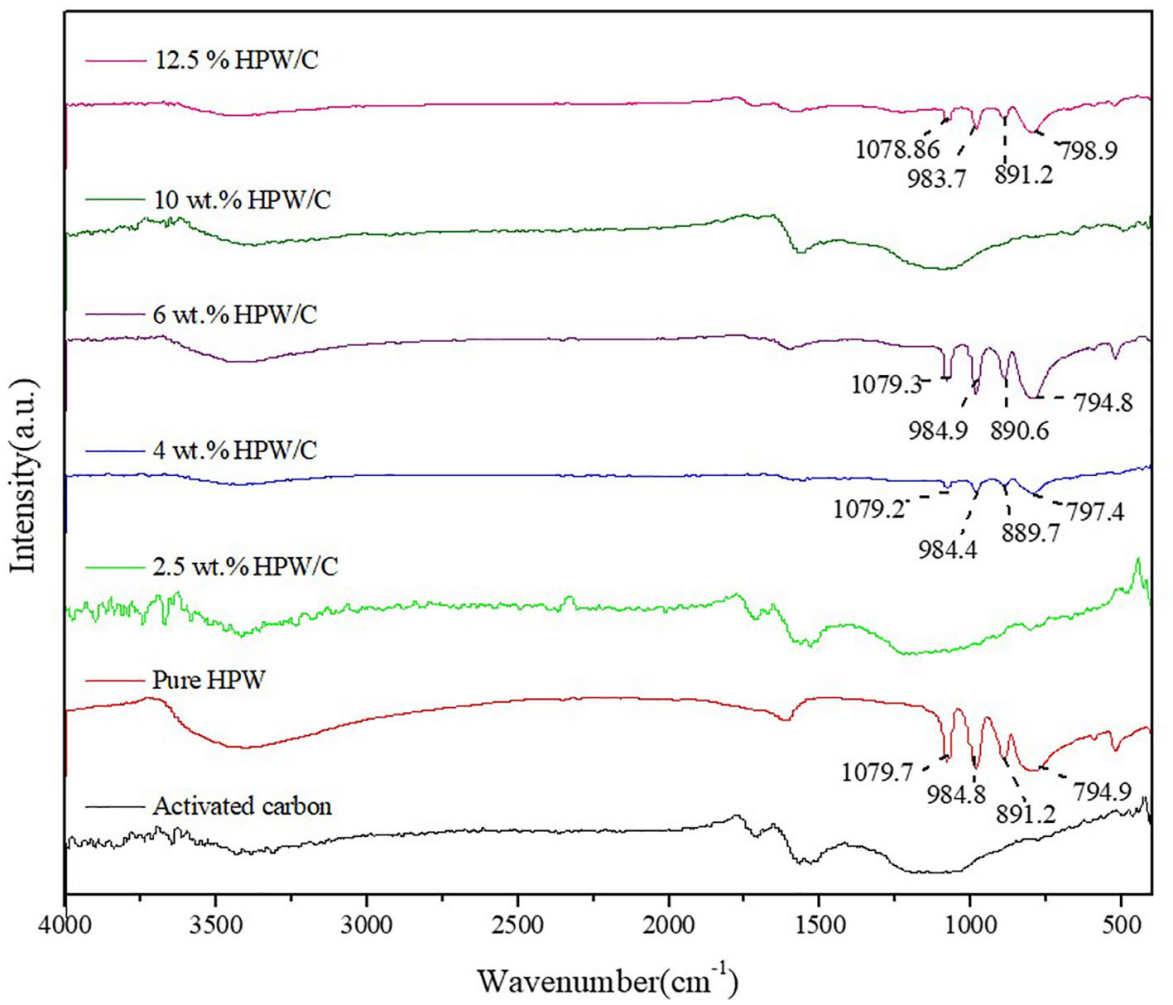
The FTIR pattern of activated carbon, HPW and catalysts samples with different HPW loadings.

Figure 5 showed the infrared spectra of different supported catalysts and pure catalysts. It can be seen that the characteristic peaks of the Keggin structure still appear after HPW was loaded on activated carbon, which indicated that the Keggin structure of HPW had not changed after activated carbon, but some characteristic peaks had shifted. The vibration of bridge oxygen W-O_c_-W was blue shifted, the terminal oxygen W = O_d_ vibration was red shifted, and the vibration of tetrahedral oxygen P-O_a_ and bridge oxygen W-O_b_-W did not shift significantly. Because the surface of activated carbon contains a large number of oxygen-containing groups, such as carboxyl, hydroxyl, phenol, and carbonyl groups, HPW will interact with these oxygen-containing groups during the adsorption process (Timofeeva et al., 2004). The terminal oxygen W = O_d_ and bridge oxygen W-O_c_-W were outside the Keggin anion and were directly bonded to the oxygen-containing group, which caused the IR characteristic peak to shift. Tetrahedral oxygen P-O_a_ and bridging oxygen W-O_b_-W were inside the Keggin anion and did not interact directly with oxygen-containing groups, so no significant shift occurred (Zhang and Shunhe, 2005). It is worth noting that when the load is low, like 2.5 wt.%HPW/C, there is not the characteristic peak of HPW, however, when the load is high, the offset decreases, because under high load, the HPW that forms multi-molecular adsorption does not directly interact with the oxygen-containing groups on the support surface, and its infrared characteristic peak is close to the pure HPW. This phenomenon supports the aforementioned theory of HPW adsorption on activated carbon. But for 10 wt.%HPW/C, the characteristic peak of HPW can't be found, speculating the HPW basically entering the pore of activated carbon resulting in this phenomenon. The surface area and pore volume of 10 wt.%HPW/C also can explain that.

### Performance of Catalysts

**Figure 6:**
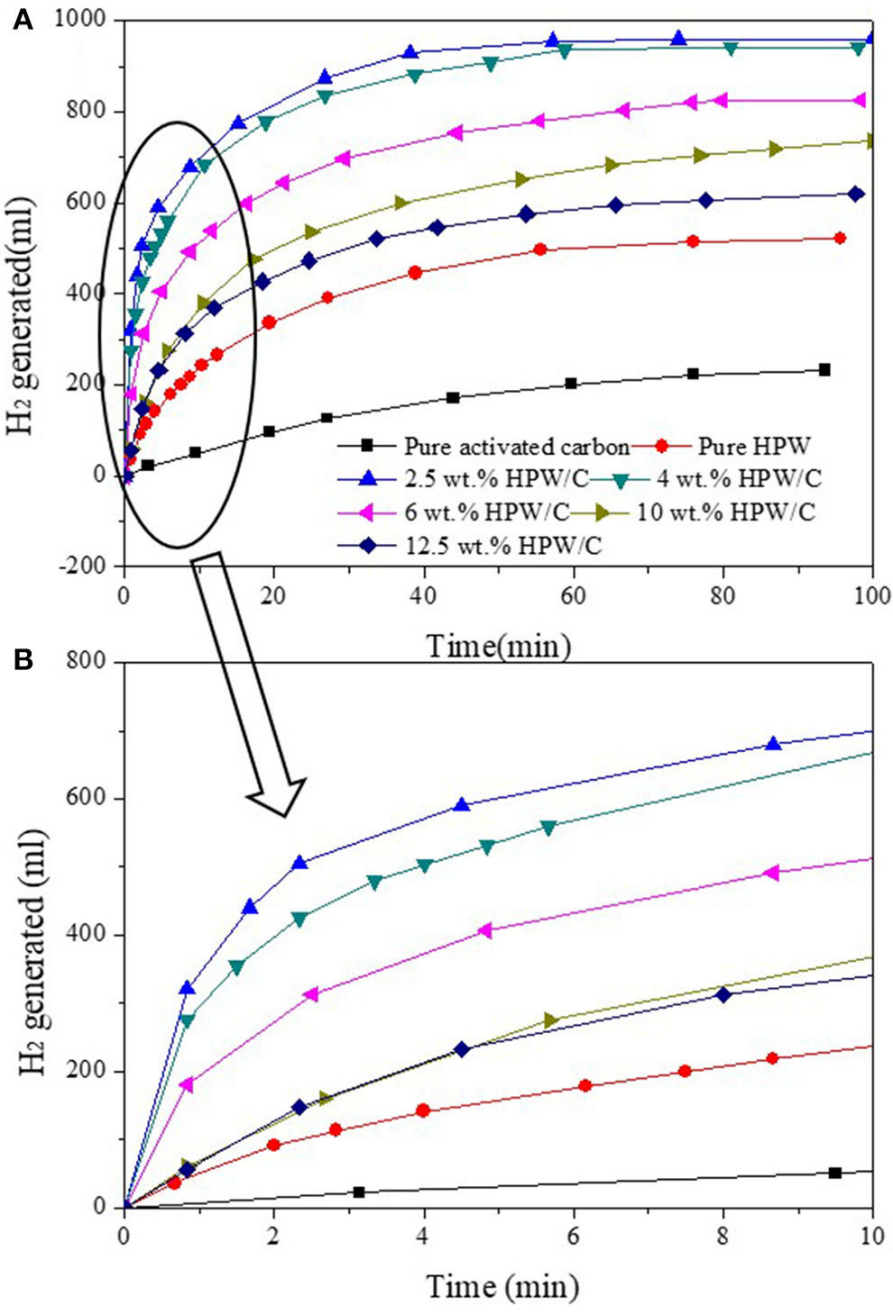
The images of NaBH4 hydrolyzed seawater with catalysts samples with different HPW loadings **(A)** and partial enlargement in 10min **(B)**.

The kinetics of NaBH_4_ hydrolysis catalyzed by catalysts were compared in Figure 6. And the hydrogen production volume and maximum hydrogen production rate of NaBH_4_ hydrolysis catalyzed by different catalysts were shown in Table 2. Compared with pure HPW, catalysts HPW/C can improve the rate of hydrogen production obviously. Nevertheless, as the increase of HPW loading on activated carbon, the rate of hydrogen production decreases. The reason for this result was that excessive loading of HPW caused HPW multilayer adsorption on activated carbon, which may cause clogging of activated carbon pores (reduce the surface area, see Table 1), which cannot make HPW fully contact with the reaction solution and NaBH_4_, thus reducing the catalytic effect.

**Table 2:** Comparison of catalytic performance between pure HPW and catalysts samples with different HPW loadings effect on seawater or DI water.

**Catalysts**	**Reaction mixture**	**Theoretical hydrogen production (ml)**	**Experimental hydrogen production (ml)**	**Maximum rate of hydrogen production [ml/(min·gcat)]**
Activated carbon	seawater	1187.1	231	140
Pure HPW	seawater	1185.2	520	1,080
2.5 wt.%HPW/C	seawater	1185.7	955	7,680
2.5 wt.%HPW/C	DI water	1197.7	850	6,800
4.0 wt.%HPW/C	seawater	1186.4	946	6,600
4.0 wt.%HPW/C	DI water	1215	880	6,370
6.0 wt.%HPW/C	seawater	1189	826	4,320
10.0 wt.%HPW/C	seawater	1184.7	736.5	1,440
12.5 wt.%HPW/C	seawater	1179.5	619	1320

**Figure 7:**
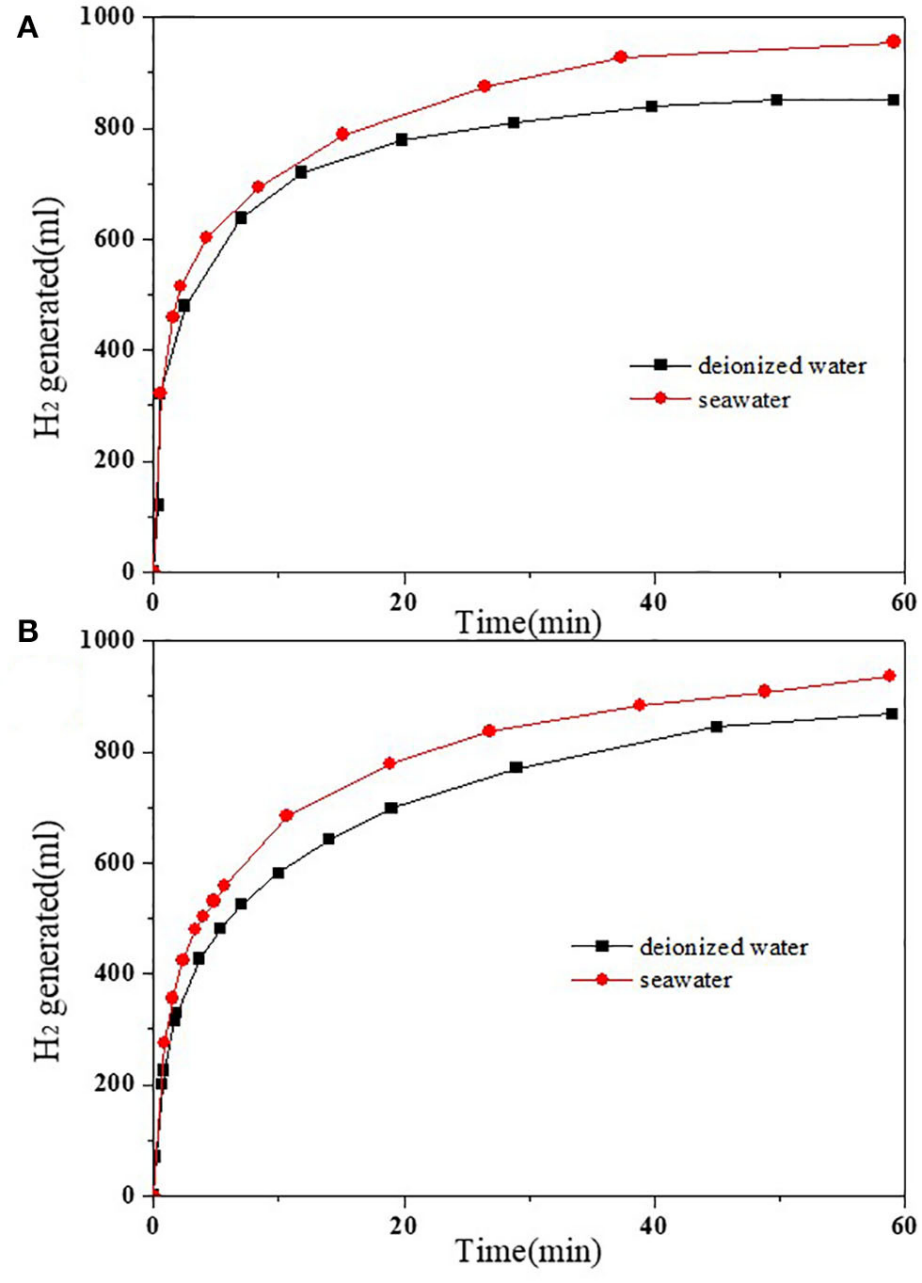
The images of NaBH_4_ hydrolyzed fresh water and seawater with 2.5 wt.%HPW/C **(A)** and 4 wt.%HPW/C **(B)**.

The best catalytic effect of 2.5 and 4 wt.%HPW/C were selected to compare the kinetics of hydrolysis of NaBH_4_ in seawater and in deionized (DI) water. The results were shown in Figure 7 and Table 2. Within 55 min, 2.5 wt.%HPW/C catalyzed NaBH_4_ hydrolysis in seawater can release 955 ml hydrogen while catalyzed NaBH_4_ hydrolysis in DI water only released 850 ml of hydrogen. And within 66 min, 4 wt.%HPW/C catalyzed NaBH_4_ hydrolysis released 946 ml of hydrogen from seawater, while catalyzed NaBH_4_ hydrolyzed and released 880 ml hydrogen from DI water. The maximum rate of NaBH_4_ hydrolyzed in seawater with catalysts 2.5 and 4 wt.%HPW/C are 7,680 ml/(min·g_cat_) and 6,600 ml/(min·g_cat_), respectively. In addition, from Figures 7A,B, the NaBH_4_ hydrolyzes faster in seawater faster than in DI water with different catalysts. Considering that seawater is alkaline and DI water is neutral, when seawater is used as the reaction liquid, the hydrolysis of HPW can be accelerated to improve the NaBH_4_ hydrolysis. And previous works of literature showed that compared with the reaction solution without NaOH, the rate of hydrogen produced by the alkaline reaction solution was faster (Sahiner et al., 2015; Singh and Das, 2017). In addition, 2.5 wt.%HPW/C was also compared with many previous works shown in Table 3, and it can still show good catalytic effect.

**Table 3:** Comparison of different catalysts' performance on the hydrolysis of sodium borohydride.

**Catalysts**	**Maximum rate of hydrogen production [ml/(min·gcat)]**	**References**
Co/Fe_3_O_4_@C	1,403	Chen et al., 2018
Co-B_bubble_	5,310	Wang et al., 2018
Acid treated sepiolite supported-CoB	1,486	Nuran et al., 2019
Intrazeolite cobalt(0) nanoclusters	6,090	Rakap and Özkar, 2009
Co/KWPA	5,884	Bennici et al., 2011
Co/CsWPA	4,039	Bennici et al., 2011
2.5 wt.%HPW/C	7,680	This work

## Conclusion

In this study, different percentage of HPW supported catalysts were prepared by impregnation method for hydrolysis of NaBH_4_. Supporting HPW on porous activated carbon can significantly increase the specific surface area of HPW, at the same time, the Keggin structure of HPW is also guaranteed, which guarantees its catalytic performance. Compared the different percentage supported catalysts on the hydrolysis of sodium borohydride in seawater, 2.5 wt.%HPW/C showed the best results. The maximum rate of hydrogen production was 7680 ml/(min·gcat). Compared the rates of hydrolysis of NaBH_4_ in seawater and in DI water with the same catalyst, the rate of NaBH_4_ hydrolyzes seawater was faster than that of DI water. When using 2.5 wt.% HPW/C and 4 wt.% HPW/C to catalyze, the maximum hydrogen production rate in NaBH_4_ in seawater was 880 ml/(min·gcat) and 230 ml/(min·gcat) faster than that in NaBH_4_ in DI water, respectively. Therefore, this study provides reliable theoretical support for seawater hydrogen production in coastal areas.

## Data Availability Statement

The raw data supporting the conclusions of this article will be made available by the authors, without undue reservation.

## Author Contributions

All authors have made substantial contributions to the concept of the work, the preparation and characterization of the materials, and the catalytic properties. The final version was revised by all authors and agreed to be published.

## Conflict of Interest

DW was employed by the company Jiangsu Xinju Environmental Co., Ltd., and XH was employed by the company Taian Boao Safety Evaluation Co., Ltd. The remaining authors declare that the research was conducted in the absence of any commercial or financial relationships that could be construed as a potential conflict of interest.
